# High mesothelin correlates with chemoresistance and poor survival in epithelial ovarian carcinoma

**DOI:** 10.1038/sj.bjc.6604964

**Published:** 2009-03-17

**Authors:** W-F Cheng, C-Y Huang, M-C Chang, Y-H Hu, Y-C Chiang, Y-L Chen, C-Y Hsieh, C-A Chen

**Affiliations:** 1 Graduate Institute of Clinical Medicine, Taipei, Taiwan; 2 Department of Obstetrics and Gynecology, College of Medicine, National Taiwan University, Taipei, Taiwan; 3 Department of Obstetrics and Gynecology, National Taiwan University Hospital, Taipei, Taiwan

**Keywords:** epithelial ovarian carcinoma, mesothelin, chemotherapy, drug resistance

## Abstract

The objective of this paper is to investigate the mesothelin expression level to the clinicopathological features, chemoresponse, and to the outcome of patients with epithelial ovarian carcinoma (EOC). Mesothelin mRNA was detected by real-time quantitative reverse-transcription PCR in 139 EOC patients. Clinical characteristics, histopathological items, responses to chemotherapy, progression-free survival (PFS), and overall survival (OS) were recorded. Tumours with advanced stages had higher mesothelin than those with early stages. The chemoresistant patients showed significantly higher mesothelin than did chemosensitive patients (2.81 *vs* 0.43, *P*<0.001), irrespective of optimal or suboptimal surgery in those with advanced stages. Highly expressed levels of mesothelin were an independent but poor prognostic factor in the PFS (2.03 (1.23–3.37) *P*=0.006) and OS (3.72 (1.64–8.45), *P*=0.002) of the 139 EOC patients in multivariate analysis. In addition, patients in advanced stages with highly expressed mesothelin also had significantly worse OS, regardless of whether they had undergone optimal (13.85 (1.76–125.60), *P*=0.013) or suboptimal (4.47 (1.83–10.88), *P*=0.001) debulking surgery in multivariate analysis. Out results provide new evidence that mesothelin expression is associated with chemoresistance and with shorter disease-free survival and worse OS of patients with EOC.

Ovarian cancer, especially epithelial ovarian carcinoma (EOC), has become an extremely important disease in recent years because it has the highest mortality rate of all gynaecological malignancies (Parkin *et al*, 1993; Beard *et al*, 2000; Boyle *et al*, 2000). Around 75% of patients are diagnosed at an advanced stage (Pfleiderer, 1984), and the overall survival (OS) rates of these patients are only 19–30% (Pfleiderer, 1984; Gonzalez-Diego *et al*, 2000). The standard treatment of EOC is surgical tumour debulking, followed by chemotherapy (Agarwal and Kaye, 2003; DiSaia and Bloss, 2003). Administration of adjuvant chemotherapy, consisting of a platinum compound (cisplatin or carboplatin), remains the standard treatment after surgery even in the early stages of EOC (DiSaia and Bloss, 2003; Schwartz, 2003; Stuart, 2003).

Platinum, combined with cyclophosphamide- or taxane-based chemotherapy, is the most common cytotoxic regimen for adjuvant chemotherapy of EOC patients (Rustin *et al*, 1996). Platinum-based chemotherapy, given at 3-week intervals for a total of three to six cycles, produces around 80% response rate in all stages of ovarian cancer (Greenlee *et al*, 2001). However, 50–70% of patients relapse and ultimately die from their cancer circumstances (Ozols, 2002). As resistance to chemotherapeutic drugs plays a major role in tumour progression (Bradshaw and Arceci, 1998; Haq and Zanke, 1998), the identification of patients who are resistant to platinum-based chemotherapy will allow the choice of cytotoxic drugs of other mechanisms, the development of novel drugs, and new therapeutic strategies.

Mesothelin is a glycoprotein to be largely restricted to mesothelial cells or to epithelial cells of the trachea, tonsils, fallopian tube, and kidneys (Chang *et al*, 1992). Mesothelin has been reported as a tumour-associated marker in several types of human cancers, including ovarian carcinomas and adenocarcinomas arising from the pancreatico-biliary tract, endometrium, and lungs (Chang *et al*, 1992). Mesothelin has also been reported to interact with CA125 to mediate cell adhesion (Rump *et al*, 2004). Although the biological functions of mesothelin remain largely unknown (Bera and Pastan, 2000), there is evidence that mesothelin has the potential as a new cancer biomarker (Cheng *et al*, 2007) and as a target molecule for gene therapy (Chang *et al*, 2007). Some investigators have reported that mesothelin can be a new marker for the diagnosis of ovarian carcinoma (Huang *et al*, 2006; Yen *et al*, 2006) and as a target in mesothelin-expressing tumours (Fan *et al*, 2002; Hassan *et al*, 2007a, 2007b).

Although mesothelin has been documented as a tumour-associated marker in EOC, analyses focussing on the correlation between mesothelin expression and clinicopathological variables and clinical outcomes have seldom been carried out. The purpose of this study is to use real-time quantitative reverse-transcription (RT)–PCR to assess the clinical significance of mesothelin expression in EOC patients.

## Materials and methods

### Patients and specimens

From July 1994 to June 2008, 139 patients with ovarian epithelial carcinoma undergoing staging or debulking surgery were enrolled. The experimental protocols were reviewed and approved by the Institutional Review Board of the National Taiwan University Hospital. Cancerous tissues were acquired after informed consent was signed. After surgical staging with debulking surgery, early- or advanced-stage patients, except those with stage IA and grade I disease, received four or six courses of adjuvant chemotherapy with platinum plus cyclophosphamide or paclitaxel regimens. The histological grading was according to the International Union against Cancer criteria (Sobin and Fleming, 1997), whereas staging was according to the criteria set by the International Federation of Gynecology and Obstetrics.

Pre-existing clinical information, including age, menopausal status, clinical stage, treatment history, surgical findings during debulking, recurrence status, and survivorship, was collected from clinical and operative notes and discharge summary that were deposited in a centralised database. The maximal diameter of the residual tumour during surgery was also recorded. Optimal debulking surgery was defined as the maximal diameter of residual tumour <1 cm or otherwise defined as suboptimal debulking surgery. Patients received regular follow-up after completion of treatment. Computerised tomography or magnetic resonance imaging was carried out when recurrence was suspected. Abnormal results of imaging studies, aspiration cytology from ascites, elevated tumour markers (⩾2-fold of upper normal limits) of two consecutive tests at 2-week intervals, or tissue proven from biopsy, if possible, were defined as recurrence. Progression-free survival (PFS) was measured as the period from operation to the date of confirmed recurrence or disease progression, or to the date of the investigators' last note of a disease-free status. Patients with disease progression or with disease recurrence ⩽6 months after discontinuing chemotherapy were defined as chemoresistant, whereas those without recurrence or with recurrence >6 months after discontinuing chemotherapy were defined as chemosensitive.

### Extraction of RNA in ovarian cancer tissues

Cancerous tissue specimen was collected during surgery, was immediately frozen in liquid nitrogen, and stored at −70°C until analysis. The total RNA of ovarian cancer tissues was isolated using TRIzol reagent (Invitrogen, Carlsbad, CA, USA) following the manufacturer's instructions.

### Real-time quantitative RT–PCR

Mesothelin and G6PDH RNA were first reverse transcribed to cDNA. Real-time PCR was carried out using the LightCycler Real-Time detection system (Roche Diagnostics, Mannheim, Germany), according to the manufacturer's protocol, for 50 cycles of 10 s at 95°C, 10 s at 60°C, and 10 s at 72°C. 5′-CTATTCCTCAACCCAGATGCGT and 3′-GCACATCAGCCTCGCTCA were the primers used to detect mesothelin. The detection of G6PDH was carried out by the LightCycler h-G6PDH housekeeping gene set (Roche Applied Science, Indianapolis, IN, USA) for 50 cycles of 10 s at 95°C; 15 s at 55°C, and 15 s at 72°C. Generation of quantitative data is based on the number of cycles needed for amplification-generated fluorescence to reach a specific threshold of detection (the *C*t value). For the relative quantification of gene expression on the basis of adding fixed amounts of RNA-starting material to the reactions, the *C*t values obtained for each real-time PCR were first transformed using the term *E*^−*C*t^, where *E*=reaction efficiency, and then divided by the corresponding value obtained for the same gene in the reference sample (normal ovarian tissues). To obtain the (ΔΔ*C*t) value, we first quantified the Δ*C*t value as indicated: *C*t_target (mesothelin)_−*C*t_housekeeping (G6PDH)_, then ΔΔ*C*t would be obtained as Δ*C*t_sample (ovarian cancer tissue)_−Δ*C*t_calibrator (normal ovarian tissue)_. The comparative 2^−ΔΔ*C*t^ method was carried out as described earlier (Livak and Schmittgen, 2001). The equation to calculate the expression level of mesothelin in each sample is as follows:

Relative expression level of mesothelin=2^−ΔΔ*C*t^, Δ*C*t=*C*t_target_−*C*t_housekeeping_, ΔΔ*C*t=Δ*C*t_cample_−Δ*C*t_calibrator_.

### Quantitative analysis of mesothelin by semi-quantitative RT–PCR

RNA was first reverse transcribed to cDNA using the Moloney murine leukaemia virus reverse transcriptase kit (Invitrogen Life Technologies, San Diego, CA, USA). For the generation of mesothelin, a set of primers, 5′-TTGTGCCCACTTCTTCTCCCTCA-3′ and 5′-CTCATCCAACACTGCTACCAAGC-3′, for 30 cycles was used. Glyceraldehyde-3-phosphate dehydrogenase (GAPDH) was used as the housekeeping gene to compare with our target gene, mesothelin. For the generation of GAPDH, a set of primers, 5′-ACCCAGAAGACTGTGGATGG-3′ and 5′-TGCTGTAGCCAAATTCGTTG-3′, for 23 cycles was also used.

The products of PCR were then analysed in 1% agarose gel with ethidium bromide staining in TBE solution. PCR products of around 440 and 520 bp were regarded as exact products and periodic DNA sequencing was carried out to confirm whether the PCR products were exact. The gel images were then obtained by using a CCD camera (Biocapt company, Vilbert Lourmat, Marne la Vallée, France) and the bands of interest were stored as TIFF files using BioCapt software as described earlier (Masson *et al*, 2001). The density of mesothelin/density of GAPDH was regarded as the expression level of mesothelin in each ovarian cancer tissue.

### Statistical analysis and clinical correlation

Statistical analyses were carried out with the Statistical Package of Social Studies (SPSS) version 8.0 (SPSS Inc., Chicago, IL, USA) for Windows. Comparisons between unpaired groups were made using the Mann–Whitney *U* test, one-way analysis of variance (ANOVA), Student's *t*-test, the *χ*^2^ test, and the Kruskal–Wallis *H* test. Spearman's correlation was used to determine the association between RTQ RT–PCR and semi-quantitative RT–PCR (SQ RT–PCR) values in mesothelin expression.

Serum CA125 and mesothelin expression levels were first assessed as continuous variables and analysed using the Mann–Whitney *U* test and the Kruskal–Wallis *H* test. The serum CA125 and mesothelin expression levels were further assessed as categorical variables. The values of serum CA125 were divided at 1000 U ml^−1^ and the values of mesothelin expression levels >1 after an earlier calculation were defined as highly expressed and those <1 as low expressed for the survival analyses. Survival curves were generated using the Kaplan–Meier method and differences in survival curves were calculated using the log rank test. Cox's univariate and multivariate regression analyses were used to evaluate the prognostic factors for PFS and OS survival. A *P*-value less than 0.05 was considered statistically significant.

## Results

The clinicopathological items between the chemosensitive and chemoresistant groups were also shown in Table 1. Chemoresistant patients were older, had larger amounts of ascites, higher percentage of advanced stages, and higher incidence of suboptimal debulking surgery than did chemosensitive patients. However, the number of gravida and parity, status of menopause, histological types, tumour histological grading, lymph node metastasis, and pre-operative serum CA125 levels were not different between the two groups.

The clinicopathological features and pre-operative CA125 serum levels of the enrolled patients are shown in Table 2. The median age was 54 years (range: 28–80 years) at the time of diagnosis. The median duration of follow-up was 21 months (range: 1–102 months) and median disease-free interval was 11 months (range: 0–80 months), with 54 and 25 patients having recurrent and persistent diseases, respectively. Thirty-nine (28.1%) died, 31 (22.3%) lived with the disease, and 69 (49.6%) were disease free. Patients with serous, endometrioid, or mixed histological types, with advanced stages, and with suboptimal debulking surgery had significantly higher pre-operative serum levels of CA125 as compared with those with mucinous or clear cell histological type, with early stages, and with optimal debulking surgery.

**Figure 1:**
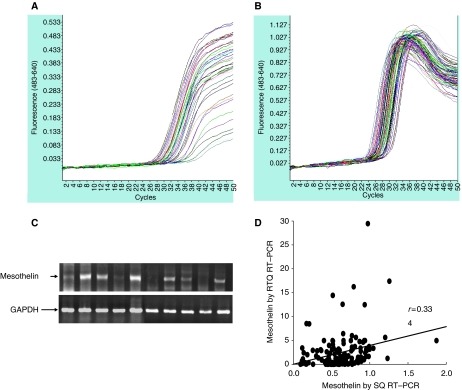
(**A**) Representative figure of quantification of mesothelin mRNA expression in tumour samples by RTQ RT–PCR. (**B**) Representative figure of quantification of G6PDH mRNA expression in tumour samples by RTQ RT–PCR. (**C**) Representative figure of mesothelin and GAPDH mRNA expressions by RT–PCR. (**D**) Correlation of mesothelin mRNA expression between RTQ RT–PCR and SQ RT–PCR (*r*=0.334).

The representative figures of RTQ RT–PCR for mesothelin (Figure 1A), RTQ RT–PCR for G6PD (Figure 1B), and SQ RT–PCR for mesothelin and GAPDH (Figure 1C) mRNA expressions are shown to evaluate the expression of mesothelin in cancerous tissues. Correlations between RTQ RT–PCR and SQ RT–PCR values for the expression of mesothelin are significantly high in this survey (*r*=0.334, *P*<0.001, Spearman's correlation) (Figure 1D).

**Figure 2:**
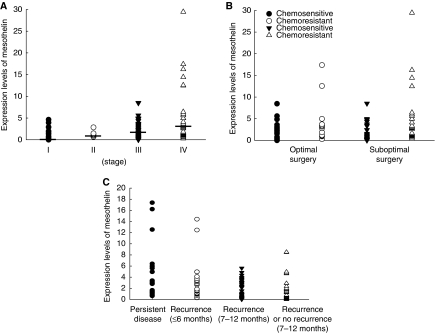
(**A**) Mesothelin mRNA expression levels between different stages of ovarian carcinoma. (**B**) The expression levels of chemosensitive and chemoresistant groups in 139 ovarian cancer patients with optimal (*n*=78) and suboptimal (*n*=61) debulking surgery. (**C**) Mesothelin mRNA expression levels in ovarian cancer patients with various progression-free intervals.

The relationships between clinicopathological items and expression levels of mesothelin were further evaluated. Patients with advanced stages (stage I: 0.31 (95% CI 0.08–1.52), stage II: 0.67 (95% CI 0.12–1.67), stage III: 1.52 (95% CI 0.33–3.35), stage IV: 2.60 (95% CI 0.94–4.96), *P*=0.005), with high histological grade (poorly differentiated) (grade 1: 0.65 (95% CI 0.04–1.24), grade 2: 0.65 (95% CI 0.05–2.81), grade 3: 1.65 (95% CI 0.43–3.39), *P*=0.015), and with suboptimal debulking surgery (optimal: 0.92 (95% CI 0.12–2.71), suboptimal: 1.54 (95% CI 0.55–4.35), *P*=0.021) had significantly higher mesothelin as compared with those with early stages, lower histological grades, and with optimal debulking surgery (Table 2). Figure 2A showed the expression levels of mesothelin in different stages.

The chemoresistant patients showed significantly higher mesothelin than the chemosensitive patients (2.81 (95% CI 1.16–4.96) *vs* 0.43 (95% CI 0.06–2.13), *P*<0.001). We then divided the patients into four subgroups, such as patients without recurrence or with recurrence >12 months after chemotherapy (*n*=49), those with recurrence within 6–12 months after chemotherapy (*n*=38), those with recurrence <6 months after chemotherapy (*n*=27), and those with persistent disease (*n*=25). As shown in Figure 2B, the mesothelin in the group with persistent disease and with recurrence <6 months after chemotherapy was significantly higher than that in patients with recurrence within 7–12 months after chemotherapy, and without recurrence or with recurrence >12 months after chemotherapy (*P*<0.001, one-way ANOVA). However, the mesothelin expression was not different between groups of patients without recurrence or with recurrence longer than 12 months after chemotherapy and between patients with recurrence within 6–12 months after chemotherapy (*P*>0.05, one-way ANOVA).

As advanced stages and residual tumour volume have been shown to correlate with the chemoresponse of EOC patients, there was additional focus on the 104 advanced-stage patients, who were divided into two groups – those with and those without optimal debulking surgery – to evaluate the correlations between mesothelin expression and chemoresponse. The chemoresistant group showed significantly higher mesothelin than did the chemosensitive group, regardless of optimal (*n*=44) (2.99 *vs* 1.17, *P*<0.001) or suboptimal (*n*=60) (3.12 *vs* 0.34, *P*<0.001) debulking surgery (Figure 2C).

Probable factors in clinicopathological parameters were then evaluated and biomarkers were evaluated to predict PFS for the 139 EOC patients (Table 3). Advanced stages (stage III/IV *vs* I/II, 1.84 (1.32–2.57), *P*<0.001), without optimal debulking surgery (no *vs* yes, 2.36 (1.50–3.70), *P*<0.001), time to normal CA125 level cut-off within 63 days (longer *vs* less, 3.45 (2.17–5.47), *P*<0.001), and expression levels of mesothelin (high *vs* normal, 2.63 (1.63–4.26), *P*<0.001) showed significantly shorter PFS as compared with early stages, undergoing optimal debulking surgery, time to CA125 level less than 63 days, and normal expression levels of mesothelin by univariate analysis. However, highly expressed levels of mesothelin (2.03 (1.23–3.37), *P*=0.006) and time to normal CA125 level (cut-off within 63 days) (2.14 (1.28–3.58), *P*=0.004) were two independent and poor prognostic factors in the PFS of the 139 EOC patients in multivariate analysis.

**Figure 3:**
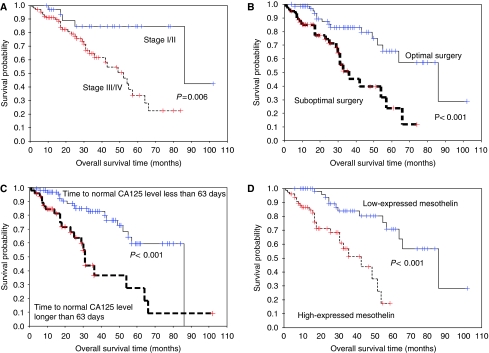
(**A**) The overall survival of 139 ovarian carcinoma patients with early and advanced stages (*P*=0.006). (**B**) The OS of 139 ovarian carcinoma patients with optimal and suboptimal debulking surgery (*P*<0.001). (**C**) The OS of 139 ovarian carcinoma patients with time to normal CA125 level less and longer than 63 days (*P*<0.001). (**D**) The OS of 139 ovarian carcinoma patients with high- and low-expressed mesothelin levels (*P*<0.001).

Probable factors in clinicopathological parameters and biomarkers were further calculated to predict OS. As shown in Table 4, the stage (stages III/IV *vs* I/II, 2.08 (1.24–3.50), *P*=0.006) (Figure 3A), optimal debulking surgery (no *vs* yes, 3.35 (1.71–6.54), *P*<0.001) (Figure 3B), time to normal CA125 level cut-off within 63 days (longer *vs* less, 3.41 (1.80–6.47), *P*<0.001) (Figure 3C), and expression levels of mesothelin (high *vs* normal, 4.64 (2.15–10.03), *P*<0.001) (Figure 3D) showed significantly shorter OS by univariate analysis. However, highly expressed mesothelin (3.72 (1.64–8.45), *P*=0.002) was the only independent and poor prognostic factor for OS by multivariate analysis.

**Figure 4:**
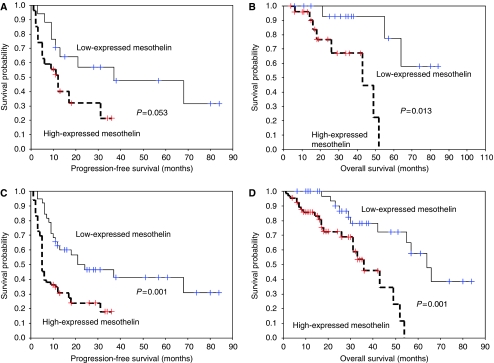
(**A**) The progression-free survival (PFS) of 44 advanced-stage ovarian cancer patients undergoing optimal debulking surgery with high- and low-expressed mesothelin (*P*=0.053). (**B**) The overall survival of 44 advanced-stage ovarian cancer patients undergoing optimal debulking surgery with high- and low-expressed mesothelin levels (*P*=0.013). (**C**) The PFS of 60 advanced-stage ovarian cancer patients undergoing suboptimal debulking surgery with high- and low-expressed mesothelin levels (*P*=0.001). (**D**) The OS of 60 advanced-stage ovarian cancer patients undergoing suboptimal debulking surgery with high- and low-expressed mesothelin levels (*P*=0.001).

The expression level of mesothelin was evaluated if it could also be a prognostic factor for the PFS and OS of the advanced-stage EOC patients with or without optimal debulking surgery. The group of highly expressed mesothelin showed marginally shorter PFS (2.43 (0.99–5.95), *P*=0.053) and significantly worse OS (13.85 (1.76–125.6), *P*=0.013) as compared with that of low-expressed mesothelin in 44 patients in advanced-stage EOC with optimal debulking surgery (Figure 4A and B). The group of highly expressed mesothelin showed significantly shorter PFS (2.48 (1.44–4.27), *P*=0.001) and worse OS (4.47 (1.83–10.88), *P*=0.001) as compared with that of low-expressed mesothelin in 60 patients of advanced-stage EOC with suboptimal debulking surgery (Figure 4C and D).

## DISCUSSION

Mesothelin may be an emerging marker for diagnosis and target-based therapy in ovarian epithelial cancers. Ovarian cancer is one of the most common types of carcinoma that overexpresses mesothelin. Our SQ RT–PCR and QRT RT–PCR analyses confirmed earlier reports (Chang and Pastan, 1996; Schaner *et al*, 2003) that mesothelin was expressed in cancerous tissues of ovarian carcinomas, suggesting that mesothelin could be an ideal marker for cancer diagnosis and target-based therapy. Recently, several molecules other than mesothelin have been found to be potential biomarkers for ovarian carcinoma, such as DF3, vascular endothelial growth factor, MUC1, HE4, and CA19-9 (Cheng *et al*, 1999; Rosen *et al*, 2005; Hefler *et al*, 2006). Moore *et al* (2008) also reported that combined CA125 and HE4 is a more accurate predictor of malignancy than either of them alone. The challenge is to conduct a prospective study using comprehensive gene expression analyses, including these potential biomarkers with sufficient patient numbers.

The first significant finding of this study is that mesothelin expression conferred a poorer chemoresponse in EOC patients. As the effect of cytotoxic drugs is influenced by histological stage and tumour volume, we analysed if the expression level of mesothelin could correlate with the chemoresponse of EOC patients with similar histological stages and residual tumour sizes. The expressions of mesothelin in chemosensitive EOC patients with residual tumour size ⩽1 or >1 cm were significantly lower than those in the chemoresistant groups, which indicates that mesothelin can be a potential biomarker to evaluate chemotherapeutic effects on EOC patients.

Our findings imply that cancer cells containing greater levels of mesothelin can resist cytotoxic drug-induced apoptosis and will continue to progress, unlike other tumour cells that fail to express mesothelin. Mesothelin may have the function of regulating the traffic of molecules and cells into and out of the peritoneal cavity (Bera and Pastan, 2000). We hypothesise that mesothelin may alter the time spent by cytotoxic drugs in the peritoneal cavity, or change the tumour microenvironment of ovarian cancer patients so as to inhibit the effects of cytotoxic drugs. Nonetheless, other mechanisms may also exist although the major mechanisms of resistance that have been identified thus far involve reduced drug uptake, increased drug efflux, increased repair of platinum-DNA adducts, increased tolerance of DNA damage, and increased levels of intra-cellular thiols, such as glutathione and metallothionein.

Biomarkers to predict the chemotherapeutic response have clinical significance in the management of EOC patients (Kupryjanczyk *et al*, 2003; de Graeff *et al*, 2008). Adjuvant chemotherapy has improved disease-free intervals and OS in various malignancies. However, the treatment can still be viewed largely as a ‘shot in the dark’ and the tools available to help predict who will respond optimally to which treatment are still relatively crude. Some molecules, such as BRCA1 (Quinn *et al*, 2007), soluble Fas levels (Chaudhry *et al*, 2008), Death Receptor 4 and TNF receptor 2 (TNFR2) (Dong *et al*, 2008), EF24 (Selvendiran *et al*, 2007), and trophinin (Baba *et al*, 2007), have been shown to correlate with cisplatin resistance in human ovarian cancer. The molecular markers involved in the activity of chemotherapeutic agents can shed light on the successes and failures of treatment in ovarian cancer patients and can also provide a basis for individualised therapy.

The second significant finding of this study is that mesothelin expression confers poor clinical outcome in EOC patients. The correlation of mesothelin expression with several other clinicopathological features, including pathological stage, tumour grade, drug resistance status, and status of optimal surgery, indicates that mesothelin seems to be associated with these variables. The Kaplan–Meier analysis for 104 stage III–IV cases revealed that the PFS and OS of 33 patients of highly expressed mesothelin were 6 *versus* 15 months (*P*=0.026) and 18 *versus* 28 months (*P*=0.008), respectively. There was no significant difference between these groups in age, tumour histology, tumour debulking status, or chemotherapeutic regimens, which shows that mesothelin is overexpressed in tumours with worse outcome and is suggestive of its independently preventive role in ovarian tumour progression.

Shih and co-workers reported that high-grade ovarian serous adenocarcinoma patients with diffuse mesothelin staining had better median OS than those with negative or focal mesothelin staining by the distributing pattern of mesothelin (Yen *et al*, 2006). The mesothelin expression and OS of 59 ovarian high-grade serous adenocarcinoma were also analysed in this study. The 42 mesothelin highly expressed, high-grade serous adenocarcinoma patients showed a marginally shorter OS than did 17 mesothelin low-expressed patients (13 *vs* 34 months, *P*=0.055). We also carried out immunostaining for some of our patients. Our results revealed that the expression of mesothelin had good correlations between the intensity of immunostaining and RTQ RT–PCR values (data not shown). RTQ RT–PCR method is more objective and sensitive to quantitate the amount of mesothelin expression than the immunostaining method, though this method cannot identify the exact cells that secrete mesothelin. It is interesting to evaluate the correlation between the expression levels of mesothelin and its distributing pattern.

Our study presenting mesothelin expression in ovarian carcinomas provides new evidence that a higher mesothelin expression is associated with chemoresistance in patients and shorter patient survival. Multi-institutional studies will be required to confirm whether mesothelin is a really independent predictor for chemotherapy in EOC patients. Future gene therapy directed towards enhancing mesothelin expression in cancer cells might offer a new treatment strategy for ovarian cancer patients.

## Figures and Tables

**Table 1:** The summary of the patient characteristics, preoperative median serum levels of CA125, and expression levels of mesothelin in 87 chemosensitive and 52 resistant ovarian epithelial cancer patients

	**Total population**	**Chemosensitive**	**Chemoresistant**	***P*-value**
**Patient number**	**139**	**87**	**52**	
Age (mean±s.d.)	54.7±11.9	52.7±11.2	57.9±12.3	0.015^a^
Gravida	3.2±2.5	2.7±2.5	3.7±2.4	0.34^a^
Parity	2.2±1.8	1.9±1.6	2.5±1.8	0.43^a^
Tumour size (cm)	10.9±5.8	11.9±6.1	9.3±5.1	0.008^a^
				
*Ascites (ml)*				
Median (25th to 75th percentile)	500 (50–2500)	300 (50–1800)	1050 (100–3600)	0.013^b^
				
*Menopause*				
Yes	83 (60%)	50	33	0.49^c^
No	56 (40%)	37	19	
				
*Histology*				
Serous	86 (62%)	51	35	0.31^c^
Non-serous	53 (38%)	36	17	
				
*Stage*				
Early (I and II)	35 (25%)	30	5	0.001^c^
Advanced (III and IV)	104 (75%)	57	47	
				
*Grading*				
1 and 2	35 (25%)	30	5	0.069^c^
3	104 (75%)	57	47	
				
*Debulking surgery*				
Optimal	78 (56%)	60	18	<0.001^c^
Suboptimal	61 (44%)	27	34	
				
*Lymph node metastasis*				
Yes	30 (39%)	17	13	0.25^c^
No	46 (61%)	32	14	
				
CA125 (U ml^−1^)	1046	899	1320	
Median (25th to 75th percentile)	(265–2560)	(253–2344)	(456–4770)	0.059^b^

a Student's *t*-test.

b Mann–Whitney *U* test.

c χ^2^ test between chemosensitive and chemoresistant groups.

**Table 2:** The clinico-pathological items, preoperative median serum levels of CA125, and mesothelin expression levels in 139 ovarian epithelial carcinoma patients

	**Total population (*n*=139)**			**Chemosensitive group (*n*=87)**			**Chemoresistant group (*n*=52)**					**Chemosensitive group (*n*=87)**			**Chemoresistant group (*n*=52)**		
**Items**	**Number (%)**	**CA125 (U ml^−1^) (median) (25–75%)**	***P*-value**	**Number (%)**	**CA125 (U ml^−1^) (median) (25–75%)**	***P*-value**	**Number (%)**	**CA125 (U ml^−1^) (median) (25–75%)**	***P*-value**	**Mesothelin expression level (median) (25–75%)**	***P*-value**	**Number (%)**	**Mesothelin expression level (median) (25–75%)**	***P*-value**	**Number (%)**	**Mesothelin expression level (median) (25–75%)**	***P*-value**
*Histology*																	
Serous	86 (61%)	1320 (443–2857)	<0.001^a^	51 (59%)	1047 (443–2577)	<0.001^a^	35 (67%)	1322 (528–4996)	0.052^a^	1.54 (0.36–3.16)	<0.001^a^	51 (59%)	0.65 (0.07–2.39)	0.24^a^	86 (61%)	2.85 (2.33–4.96)	0.34^a^
Mucinous	6 (4%)	48 (39–139)		5 (6%)	53 (19–1179)		1 (2%)	34		0.93 (0.29–2.13)		5 (6%)	0.92 (0.04–2.13)		6 (4%)	3.18	
Endometrioid	17 (12%)	1012 (247–2561)		12 (13%)	767 (216–1345)		5 (10.0%)	4544 (1514–22 338)		0.73 (0.03–2.52)		12 (13%)	0.09 (0.01–1.01)		17 (12%)	1.15 (0.31–5.70)	
Clear cell	21 (15%)	90 (49–1534)		13 (15%)	75 (35–1240)		8 (15%)	401 (84–1124)		1.04 (0.22–2.96)		13 (15%)	0.40 (0.11–2.96)		21 (15%)	1.45 (0.82–4.16)	
Mixed	8 (6%)	1944 (26–2674)		6 (7%)	2151 (505–3081)		1 (2%)	8766 (1332–16 200)		0.25 (0.05–2.88)		6 (7%)	0.10 (0.02–0.58)		8 (6%)	10.59 (4.96–16.22)	
Undifferentiated	1 (1%)	769		0 (0%)			2 (4%)	769		4.96		0 (0%)	NA		1 (1%)	4.96	
																	
*FIGO stage*																	
I	25 (18%)	120 (39–446)	<0.001^a^	24 (27%)	175 (41–767)	<0.001^a^	1 (2%)	64	0.19^a^	0.31 (0.08–1.52)	0.005^a^	24 (27%)	0.22 (0.08–1.58)	0.24^a^	1 (2%)	0.68	0.17^a^
II	10 (7%)	456 (69–873)		6 (7%)	458 (69–2648)		4 (8%)	456 (220–1272)		0.67 (0.12–1.67)		6 (7%)	0.12 (0.10–1.14)		4 (8%)	1.30 (1.04–2.88)	
III	85 (61%)	1585 (685–2857)		49 (57%)	1604 (699–2545)		36 (69%)	1468 (690–5820)		1.52 (0.33–3.35)		49 (57%)	0.58 (0.36–2.37)		36 (69%)	3.14 (1.21–4.96)	
IV	19 (14%)	1199 (372–2674)		8 (9%)	1505 (355–2642)		11 (21%)	1199 (742–3216)		2.60 (0.94–4.96)		8 (9%)	1.18 (0.39–6.40)		11 (21%)	2.81 (2.13–4.95)	
																	
*Tumour grading*																	
I	19 (14%)	865 (228–1494)	0.53^a^	14 (16%)	828 (238–1485)	0.64^a^	5 (10%)	1042 (108–4419)	0.81^a^	0.65 (0.04–1.24)	0.015^a^	14 (16%)	0.12 (0.01–0.74)	0.007^b^	5 (10%)	3.18 (1.08–11.68)	0.86^a^
II	28 (20%)	1456 (265–3212)		20 (23%)	1573 (246–2557)		8 (15%	2358 (314–4770)		0.65 (0.05–2.81)		20 (23%)	0.16 (0.06–1.12)		8 (15%	2.98 (2.06–3.91)	
III	92 (66%)	1199 (312–2609)		53 (61%)	899 (238–2417)		39 (75%)	1320 (711–6332)		1.65 (0.43–3.39)		53 (61%)	1.11 (0.13–2.80)		39 (75%)	2.85 (1.18–4.95	
																	
*Optimal surgery*																	
Yes	78 (56%)	718 (142–1772)	<0.001^b^	60 (69%)	710 (142–1899)	<0.007^b^	18 (35%)	731 (282–1776)	0.23^b^	0.92 (0.12–2.71)	0.021^b^	60 (69%)	0.47 (0.07–2.14)	0.95^b^	18 (35%)	2.34 (0.97–3.56)	0.022^b^
No	61 (44%)	1689 (715–3212)		27 (31%)	1743 (660–2521)		34 (65%)	1437 (715–6484)		1.54 (0.55–4.35)		27 (31%)	0.34 (0.06–1.97)		34 (65%)	3.01 (1.39–4.96)	
																	
*Chemoresponse*																	
Sensitive	87 (63%)	899 (253–2344)	0.059^b^	NA	NA	NA	NA	NA	NA	0.43 (0.06–2.13)	<0.001^b^	NA	NA	NA	NA	NA	NA
Resistant	52 (37%)	1320 (456–4770)								2.81 (1.16–4.96)							

Abbreviation: NA=not available.

a Kruskal–Wallis *H* test.

b Mann–Whitney *U* test.

**Table 3:** Univariate and multivariate analyses of prognostic factors on the PFS of 139 ovarian epithelial carcinoma patients

	**Univariate**		**Multivariate**	
	**Odds ratio (95% CI)**	***P*-value^a^**	**Odds ratio (95% CI)**	***P*-value^a^**
*Histology*				
Serous/non-serous	1.46 (0.91–2.35)	0.12		
				
*Stage*				
III and IV/I and II	1.84 (1.32–2.57)	<0.001	1.38 (0.95–2.02)	0.094^b^
				
*Grade*				
3/1 and 2	1.21 (0.95–1.54)	0.12		
				
*Optimal debulking surgery*				
No/yes	2.36 (1.50–3.70)	<0.001	1.37 (0.82–2.29)	0.23^b^
				
*Lymph node metastasis*				
Yes/no	1.56 (0.83–2.92)	0.17		
				
CA125 levels>/⩽1000 U ml^−1^	1.34 (0.86–2.09)	0.21		
				
*Time to normal CA125 level*				
Longer/less than 63 days	3.45 (2.17–5.47)	<0.001	2.14 (1.28–3.58)	0.004^b^
				
*Mesothelin expression level*				
Highly *vs* low	2.63 (1.62–4.26)	<0.001	2.03 (1.23–3.37)	0.006^b^

Abbreviations: CI=confidence interval; PFS=progression-free survival.

a By Kaplan–Meier test.

b Variable included in the multivariate model.

**Table 4:** Univariate and multivariate analyses of prognostic factors on the overall survival of 139 ovarian epithelial carcinoma patients

	**Odds ratio (95% CI)**	***P*-value^a^**	**Odds ratio (95% CI)**	***P*-value^a^**
*Histology*				
Serous/non-serous	1.25 (0.64–2.44)	0.51		
				
*Stage*				
III and IV/I and II	2.08 (1.24–3.50)	0.006	1.55 (0.86–2.82)	0.15^b^
				
*Grade*				
3/1 and 2	1.04 (0.75–1.42)	0.83		
				
*Optimal debulking surgery*				
No/yes	3.35 (1.71–6.54)	<0.001	1.88 (0.84–4.17)	0.12^b^
				
*Lymph node metastasis*				
Yes/no	1.67 (0.62–4.49)	0.31		
				
CA125 levels >1000 U ml^−1^	1.88 (0.97–3.63)	0.06		
				
*Time to normal CA125 level*				
Longer/less than 63 days	3.41 (1.80–6.47)	<0.001	1.93 (0.88–4.22)	0.099^b^
				
*Mesothelin expression level*				
High *vs* low	4.64 (2.15–10.03)	<0.001	3.72 (1.64–8.45)	0.002^b^

Abbreviation: CI=confidence interval.

a By Kaplan–Meier test.

b Variable included in the multivariate model.
